# Organic Power Electronics: Transistor Operation in the kA/cm^2^ Regime

**DOI:** 10.1038/srep44713

**Published:** 2017-03-17

**Authors:** Markus P. Klinger, Axel Fischer, Felix Kaschura, Johannes Widmer, Bahman Kheradmand-Boroujeni, Frank Ellinger, Karl Leo

**Affiliations:** 1 Dresden Integrated Center for Applied Physics and Photonic Materials, Technische Universität Dresden, Nöthnitzer Str. 61, 01187, Dresden, Germany; 2 Center for Advancing Electronics Dresden (cfead), Technische Universität Dresden, Würzburger Str. 43, 01187 Dresden, Germany; 3 Chair for Circuit Design & Network Theory, Technische Universität Dresden, Helmholtzstr. 18, 01069, Dresden, Germany

## Abstract

In spite of interesting features as flexibility, organic thin-film transistors have commercially lagged behind due to the low mobilities of organic semiconductors associated with hopping transport. Furthermore, organic transistors usually have much larger channel lengths than their inorganic counterparts since high-resolution structuring is not available in low-cost production schemes. Here, we present an organic permeable-base transistor (OPBT) which, despite extremely simple processing without any high-resolution structuring, achieve a performance beyond what has so far been possible using organic semiconductors. With current densities above 1 kA cm^−2^ and switching speeds towards 100 MHz, they open the field of organic power electronics. Finding the physical limits and an effective mobility of only 0.06 cm^2^ V^−1^ s^−1^, this OPBT device architecture has much more potential if new materials optimized for its geometry will be developed.

Organic semiconductors have a low charge carrier mobility *μ* in comparison to inorganic semiconductors, reducing transconductance and thus switching speed in transistors. However, they offer great potential for flexible electronics due to simple processing techniques and carbon-based materials^1^,^2^,^3^,^4^. Nevertheless, for most applications, device speed and driving currents need to be raised significantly, while keeping low-cost processing without high-resolution structuring. While common organic field effect transistors reach very high current densities of a few 10 kA cm^−2^ in the channel region^5^, the areal current densities remain comparably low. Optimized transistors with short channel lengths reach current densities of 1 to 20 A cm^−2^ and a transit frequency normalized to the operation voltage of about 1 to 2 MHz V^−1^, cf. ref. 6, 7, 8, 9. One prominent exception is realized by Münzenrieder *et al*. fabricating an inorganic thin-film transistor with a footprint current density of ca. 500 A cm^−2^ and a transit frequency of 135 MHz at 2 V, still meeting the requirements of plastic electronics, e.g. flexibility^10^. For comparison, modern silicon based MOSFETs easily achieve more than 1 MA cm^−2^ and transit frequencies greater than 100 GHz, cf. ref. 11. However, it has been shown that organic semiconductors are capable to sustain enormous current densities in the vertical direction (perpendicular to the substrate) towards kA cm^−2^ for diodes^12^,^13^ and up to MA cm^−2^ for simple devices with thin single layers^14^. Here, we show that these vertical operation concepts can also be used to realize transistors with outstanding properties. We demonstrate an organic permeable-base transistor (OPBT) which basically resembles the layer stack of a vertical diode with an embedded base electrode in the middle of the stack to control the current flow perpendicular to the substrate. The base electrode contains nano-holes that make it permeable for charge-carriers and allows for high current densities^15^,^16^. We demonstrate here transistors with record high current densities above 1 kA cm^−2^ at low voltages, accompanied by excellent high-frequency characteristics.

## Results

### Transistor setup

**Figure 1:**
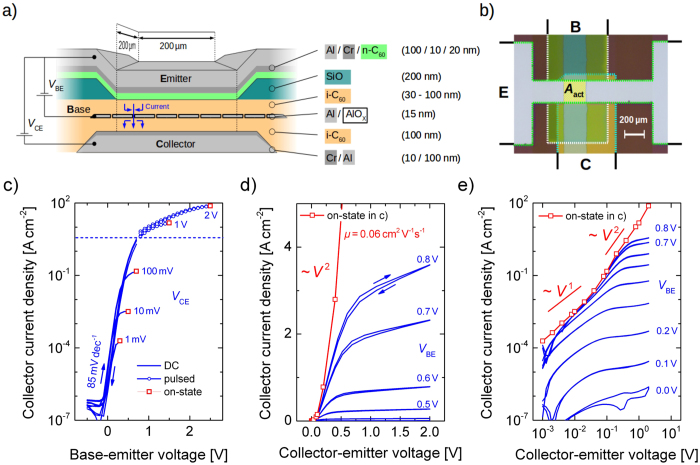
Transistor setup and DC performance. (**a**) Device cross-section and electric circuit in common-emitter configuration. Materials: aluminum (Al), chrome (Cr), n-doped C^60^ (n-C^60^), intrinsic/undoped C^60^ (i-C^60^), native aluminum-oxide (AlO_X_). Blue arrows indicate the electron flow. (**b**) Microscope top-view image showing electrode and structuring orientation as well as the active area *A*_act_ (yellow). (**c**) Transfer curves (i-C_60_: top 30 nm/bottom 100 nm) for different operation voltages *V*_CE_. A high on-off ratio of 10^8^, an on-state current density of 75 A cm^−2^ and subthreshold slope of 85 mV dec.^−1^ are achieved at a V_CE_ of 2 V. (**d**) Linear and (**e**) double logarithmic output characteristic. All curves are limited by a linear law at low voltages and a quadratic law at higher voltages, corresponding to the charge transport through the intrinsic C_60_ layer which restricts the maximum current of the transfer curve as well.

The basic OPBT setup, as shown in Fig. 1a and b, consists of three electrodes emitter, base, and collector embedding two semiconducting layers. The thin aluminum layer in the middle is passivated by a native oxide and controls the charge flow through its nano-size openings by the applied potential^17^. A detailed description of the operation mechanism can be found in ref. 16. Our results are based on an OPBT configuration as introduced in ref. 18 using C_60_ as the semiconductor material and an optimized device structuring leading to a state-of-the-art performance as presented in ref. 15. A narrowed top electrode with a width of about 200 μm is used in combination with a structured insulator-window with the same broadness to realize an active area *A*_act_ of measured 0.046 mm^2^ by thermal evaporation and low-resolution shadow masks, compatible with OLED display fabrication technology^19^.

### DC performance

Figure 1c shows the transfer curves of an OPBT for different operation voltages *V*_CE_ applied between collector and emitter. The OPBT operates even at very low voltages of 1 mV still showing an on-off ratio greater than 10^2^. However, when *V*_CE_ is higher than 1 V, the current easily reaches a level at which Joule self-heating starts, which is why the source-measuring unit is set to pulse all voltages for currents above 1 mA (>2.2 A cm^−2^). Effects of self-heating in a similar device geometry have been previously investigated^13^,^20^ and will follow for OPBTs, especially as time-resolved pulsed measurements predict that the self-heating is not completely suppressed at higher power input (cf. Supplementary Information). The OPBT has a current density of 75 A cm^−2^, an on-off ratio of 10^8^, a sub-threshold slope of 85 mV dec.^−1^ close to the theoretical optimum of 60 mV dec.^−1^, and a clockwise hysteresis below 75 mV at a moderate *V*_CE_ of 2 V. Gains greater than 10^4^ are observed (cf. Supplementary Information)^21^. The output characteristic in Fig. 1d shows a clear saturation behavior, although the channel length of this device is in the nm-range^16^. Typically, such short channel devices lose their saturation behavior due to short channel effects. We explain this effect by nano-size openings in the base electrode which screens the electric field between emitter and collector to an extent that a partial saturation can take place^22^,^23^.

### Space-charge limited current

Interestingly, the transfer curves, in Fig. 1c, reach a saturation when the base-emitter voltage *V*_BE_ gets close to *V*_CE_ indicated by red squares. Compared to the output characteristic, cf. Fig. 1d and e, these on-state currents correspond to a linear and a quadratic limit at low and a higher *V*_CE_, respectively, typically observed for diodes having a space-charge limited current (SCLC)^24^. This space charge effect in the intrinsic layers is further supported by drift-diffusion simulations in ref. 16, revealing exactly the above mentioned voltage dependence when charge injection can be considered to be ideal. Here, it is a significant advantage of the OPBTs sandwich geometry that a doped charge injection layer can be easily inserted. We use an n-doped C_60_ layer (n-C_60_), as visualized in Fig. 1a, to reduce the contact resistance that arises from the interface between the emitter electrode and the upper semiconductor layer^25^.

**Figure 2:**
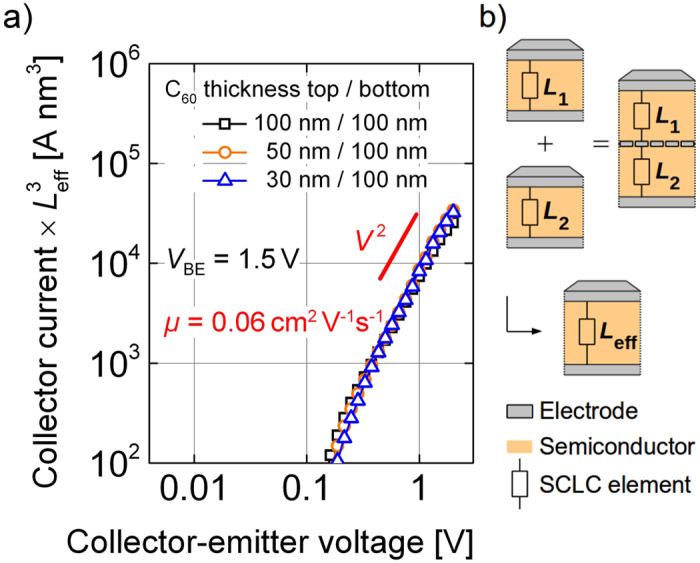
(**a**) Output characteristics at a *V*_BE_ of 1.5 V for different C_60_ layer thicknesses are normalized by the third power of the effective device length L eff to prove space-charge limited currents in OPBTs. A bulk mobility perpendicular to the substrate of 0.06 cm^2^ V^−1^s^−1^ is extracted. (**b**) Visualization of the reduced *L*_eff_ as the sum of two SCLC devices connected in series.

To show that our upper current limit is given by SCLC, the corresponding Mott-Gurney law


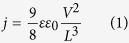


is used to describe the *L*-dependence (*ε* and *ε*_0_ are related to the permittivity)^26^. We investigate samples of different intrinsic layer thicknesses on top of the base, while the layer underneath the base remains constant so that the growth and the morphology of the base electrode can be considered to be unaffected. Figure 2a presents the output characteristic in double-logarithmic scale, each taken at a *V*_BE_ of 1.5 V. The currents scale with a cubic thickness dependence as predicted in Eq. 1. However, neither the total thickness of both intrinsic layers nor the thickness of the top intrinsic layer can be used to bring all curves together. Instead, a reduced effective length *L*_eff_, lying between the two thicknesses above, has to be used to achieve a good agreement after rescaling. This behavior can easily be understood by considering that in the on-state of the OPBT, charges strongly accumulate in front of the native oxide of the base electrode, leading to a formation of a highly conductive region which can be treated as a ‘virtual contact’^16^. As sketched in Fig. 2b, the charge transport through the upper and the lower intrinsic layer behaves in each case like a single SCLC element of length *L*_1_ and *L*_2_, respectively.

If these two elements are connected in series, as realized by the virtual contact, one can show that the current-voltage characteristics still follows Eq. 1 (cf. Supplementary Information), but with


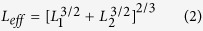


as an effective length.

These results can be concluded as follows: Firstly, the result allows to extract the bulk mobility of C_60_ for the charge transport perpendicular to the substrate of *μ* = 0.06 cm^2^ V^−1^s^−1^ (*ε* of C_60_ is ca. 4)^12^. Although the value is quite low in comparison to mobilities measured in OFETs, it allows for the excellent performance discussed above and should further motivate material scientists to put more attention on improving bulk mobilities of organic semiconductors^27^,^28^. Lateral field-effect mobilities now easily reach values up to 10 cm^2^ V^−1^s^−1^, cf. ref. 29. If it would be possible to apply this achievements to an OPBT, more than 100 times higher current densities (>1 kA cm^−2^) are possible at a low operation voltage of 1 V and a lower power dissipation. Secondly, as the major limitation is revealed to be SCLC, devices have to be as thin as possible, always chosen as a tradeoff between performance, device stability (electrical shorts), and reasonable breakdown voltages. This basically means that for a given charge carrier mobility and dimension of the semiconductor layer, the OPBT is able to drive the highest possible current densities a semiconductor can realize. All restrictions which can be seen at low voltages in the output characteristics, cf. Fig. 1d, are the restrictions which every transistor, having a vanishing channel resistance, at least has. This means, somewhat counter-intuitively, that the nano-sized openings of base electrode do not limit the charge transport. Thirdly, the voltage dependence predicts a superlinear increase when higher voltages are applied so that extreme current densities above 1 kA cm^−2^ are in reach.

### Towards 1 kA cm^−2^

To prove this implication, we change our electrode layout in a way that the emitter electrode gets wider in order to reduce its resistance. Samples with narrow emitter electrodes show a linear current-voltage relation at highest current caused by the electrode resistance of about 20 Ω and we observe that the top electrode basically rips off at the edges due to high fields and strong power dissipation (cf. Supplementary Information). When we use a wider emitter electrode, the active area of the OPBT remains constant, but is structured by two insulating layers with free stripes of 200 μm perpendicular to each other^17^.

**Figure 3:**
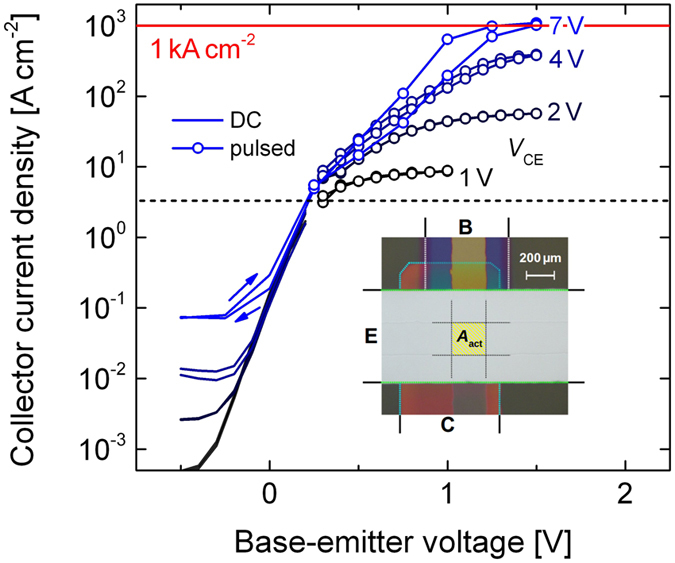
Transfer curves (i-C60: top 30 nm/bottom 50 nm) of a device with optimized, broad electrode layout at different operation voltages *V*_CE_. Applying a *V*_CE_ of 7 V leads to current densities in excess of 1 kA cm^−2^ (red line). All measurements are done with dual sweep to confirm nondestructive operation. Inset: Microscope image of the OPBT with the broaden emitter electrode.

As seen in Fig. 3, the samples show similar on-state current densities compared to the results in Fig. 1 at voltages below 2 V. The off-state current densities increase by using this structuring method, probably due to larger direct emitter-collector electrode overlap, but do not influence the characteristics of the on-state. In this way, we can prove that a current density of 1.1 kA cm^−2^ (501 mA) is driven at a *V*_CE_ of 7 V by the OPBT and a power above 1 W is applied to the structure for a short time. The non-destructive character of the measurement is proven by a dual sweep of the gate-source voltage in all cases allowing to switch from off to on-state and back, even though a hysteresis is introduced for the utmost curve. Please note that the current density in the nano-size base openings must be even much higher, estimated to be a factor of 100 to 1000 larger.

While the device cannot permanently operate at such high currents with the present thermal design of the sample, there are still cases where even our non-optimized devices can be used. For example, a transmitter circuit can radiate with an increased power, but only for a short time to decrease the power consumption of a stand-alone device. Further, selection transistors in display circuits have to be very fast, as they have to refresh each pixel at a minimum of time. I.e. high transconductance, high switching speed, and high on-off ratio are necessary, but not steady-state operation.

### AC performance

**Figure 4:**
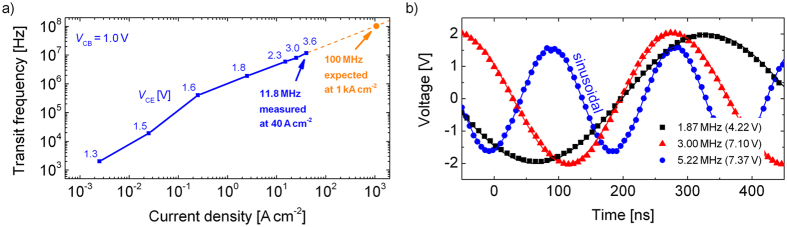
(**a**) Measured transit frequencies *f*_T_ at different current densities. An *f*_T_ up to 11.8 MHz is reached at a current density of 40 A cm^−2^. The frequency-current dependence predicts an *f*_T_ in the range of 100 MHz if a current density of 1 kA cm^−2^ would be applied. (**b**) AC-coupled performance of an OPBT in a Colpitts oscillator circuit using discrete *R-L-C* components (cf. Supplementary Information). Large-swing oscillations up to 5.22 MHz are close to an ideal sinus (fit as solid line).

The switching speed of the OPBT is investigated by measuring the transit frequency *f*_T_ at which unity current gain is reached, cf. Fig. 4a (and Supplementary Information). We use a direct-*f*_T_ measurement setup and optimized OPBTs regarding high currents^15^,^30^. At a current density *j* of 40 A cm^−2^ and a *V*_CE_ of about 3.6 V, the OPBT (top 30 nm/bottom 100 nm) amplifies signals up to 11.8 MHz, although the electrode layout is not yet optimized for reduced parasitic capacitances. Following the *f*_T_ vs. *j* characteristic, much higher *f*_T_ of about 100 MHz are expected if the current would be set to 1 kA cm^−2^. However, the *f*_T_-measurement is limited to current densities the device can withstand at steady-state conditions.

In order to demonstrate the frequency performance in a real circuit, we assemble a modified Colpitts oscillator using discrete passive *R*-*L*-*C* elements with the OPBT as active element (cf. Supplementary Information). As seen in Fig. 4b, the OPBT shows stable oscillations at frequencies of 1.87 MHz, 3.0 MHz, and 5.22 MHz when circuit supply voltages of 4.11 V, 7.10 V, and 7.37 V are applied, respectively. The signal is clearly pronounced with peak-to-peak voltages of 4 V and the output signal has a high-quality sinusoidal waveform. Oscillations of 1.87 MHz at 951 μA and 3.00 MHz at 3.2 mA are very close to the corresponding *f*_T_ shown in Fig. 4a, i.e. 1.6 MHz and 3.4 MHz, respectively. However, oscillation at 5.22 MHz is only possible at a higher current of 20 mA where the transit frequency is approximately 10 MHz. This limitation might be imposed by a non-negligible resistance of the base electrode, i.e. the internal resistor network behaves like a filter at high frequencies (cf. Supplementary Information).

## Conclusion

We present an organic permeable base transistor design which achieves an outstanding performance. The best OPBTs reach an on-off ratio of 10^8^, a subthreshold slope of 85 mV dec.^−1^ and a current density of 75 A cm^−2^ at an operation voltage of 2 V. At higher voltages, we achieve footprint current densities above 1 kA cm^−2^, introducing a new regime of operation to organic transistors. These high current densities point to transit frequencies in the 100 MHz range. The performance can even be further enhanced when the latest achievements for lateral field-effect mobilities can be transferred to vertical bulk mobilities. Thus, in future even much better vertical organic transistors could be realized with our approach.

## Methods

### Sample preparation

The OPBTs presented are built in a single chamber UHV-tool and on one glass substrate previously cleaned with N-Methylpyrrolidone, distilled water, ethanol, and Ultra Violet Ozone Cleaning System. By using thermal vapor deposition at high vacuum conditions (p < 10^−7^ mbar), the layer stack (Fig. 1a) is realized by subsequently depositing thin films through laser-cut, stainless steel shadow masks. The deposition system includes a wedge for realizing samples of different layer thickness in one run while other layers remain equal. The layer stack, evaporation rates and treatments of the OPBTs are: Al 100 nm (1 Å s^−1^)/Cr 10 nm (0.1 Å s^−1^)/i-C_60_ wedge 50, 100 nm (1 Å s^−1^)/Al 15 nm (1 Å s^−1^)/15 min oxidation at air/i-C_60_ wedge 30, 50, 100 nm/1 x or 2 x (perpendicular to each other) SiO 200 nm with a free stripe of 0.2 mm (1 Å s^−1^)/n-C_60_ 20 nm (0.4 Å s^−1^) co-evaporating C_60_ with W_2_ (hpp)_4_ (purchased from Novaled AG, Dresden) using 1 wt%/Cr 10 nm (0.1 Å s^−1^)/Al 100 nm (1 Å s^−1^)/encapsulation in a nitrogen atmosphere using UV cured epoxy glue without UV exposure of the active area/annealing for 2 h at 150 °C in a nitrogen glove-box on a heat plate. The current density of 1 kA/cm^2^ is reached for a device with a broad top electrode (600 μm) and a C_60_ bottom thickness of 50 nm and a top thickness of 30 nm. The transit frequency of 11.8 MHz is shown for an OPBT with a narrowed top electrode (200 μm) and a C_60_ bottom thickness of 100 nm and a top thickness of 30 nm. In all cases, the active area is about (200 × 200) μm^2^, realized with the support of additional insulating layers.

### Device characterization

Electrical DC-characteristics are measured with a parameter analyzer Keithley 4200-SCS, and with a source measure unit (SMU Keithley 2602A) enabling voltage-pulses with a minimum time of 250 μs. Frequency measurements are realized with an optimized measuring setup, similar to ref. 30.

## Additional Information

**How to cite this article:** Klinger, M. P. *et al*. Organic Power Electronics: Transistor Operation in the kA/cm^2^ Regime. *Sci. Rep.* **7**, 44713; doi: 10.1038/srep44713 (2017).

**Publisher's note:** Springer Nature remains neutral with regard to jurisdictional claims in published maps and institutional affiliations.

## Supplementary Material

Supplementary Information
