# Author Correction: Organic Power Electronics: Transistor Operation in the kA/cm^2^ Regime

**DOI:** 10.1038/s41598-020-63130-4

**Published:** 2020-04-10

**Authors:** Markus P. Klinger, Axel Fischer, Felix Kaschura, Johannes Widmer, Bahman Kheradmand-Boroujeni, Frank Ellinger, Karl Leo

**Affiliations:** 1 Dresden Integrated Center for Applied Physics and Photonic Materials, Technische Universität Dresden, Nöthnitzer Str. 61, 01187 Dresden, Germany; 2 Center for Advancing Electronics Dresden (cfead), Technische Universität Dresden, Würzburger Str. 43, 01187 Dresden, Germany; 3 Chair for Circuit Design & Network Theory, Technische Universität Dresden, Helmholtzstr. 18, 01069 Dresden, Germany

Correction to: *Scientific Reports* 10.1038/srep44713, published online 17 March 2017

This Article contains an error in Equation 1.

$$j=\frac{9}{8}\varepsilon {\varepsilon }_{0}\frac{{V}^{2}}{{L}^{3}}$$

should read:

$$j=\frac{9}{8}\varepsilon {\varepsilon }_{0}\mu \frac{{V}^{2}}{{L}^{3}}$$

