# Supplementary material for: Organic Power Electronics: Transistor Operation in the kA/cm2 Regime
Source: Sci Rep. 2017 Mar 17;7:44713. doi: 10.1038/srep44713 (PMC5356189; doi:10.1038/srep44713)
Supplement: Supplementary Information [file srep44713-s1.pdf]

Supplementary Information for: 'Organic Power Electronics: Transistor Operation in the kA/cm^2 Regime'

Markus P. Klinger[a], Axel Fischer[a], Felix Kaschura[a,b], Johannes Widmer[a], Bahman Kheradmand-Boroujeni[b,c], Frank Ellinger[b,c], Karl Leo[a,c]

[a] *Dresden Integrated Center for Applied Physics and Photonic Materials, Technische Universität Dresden, Nöthnitzer Str. 61, 01187, Dresden, Germany*
[b] *Chair for Circuit Design & Network Theory, Technische Universität Dresden, Helmholtzstr. 18, 01069 Dresden, Germany*
[c] *Center for Advancing Electronics Dresden (cfead), Technische Universität Dresden, Würzburger Str. 43, 01187 Dresden, Germany*

Email address: karl.leo@iapp.de (Karl Leo)

Preprint submitted to Scientific Reports *February 2, 2017*

1. Transfer curves with base current

In Figure 1 a), a base sweep is shown for an OPBT (top / bottom = 30 nm / 100 nm) including the base current. The base current (black) is several orders of magnitudes lower than the transmitted collector current (blue). The maximum of the differential transmission and the differential gain β, defined as

$$\alpha = \frac{\mathrm{d}I_C}{\mathrm{d}I_E} \quad \text{and} \quad \beta = \frac{\mathrm{d}I_C}{\mathrm{d}I_B}, \tag{1}$$

ensuring that parasitic leakage current flowing from the base to the collector leads rather to underestimated values. In Figure 1 b), a differential gain greater than 10^5 is shown. Ergo, the differential transmission reveals, that 99.999 % of the injected electrons at the emitter pass the permeable base electrode

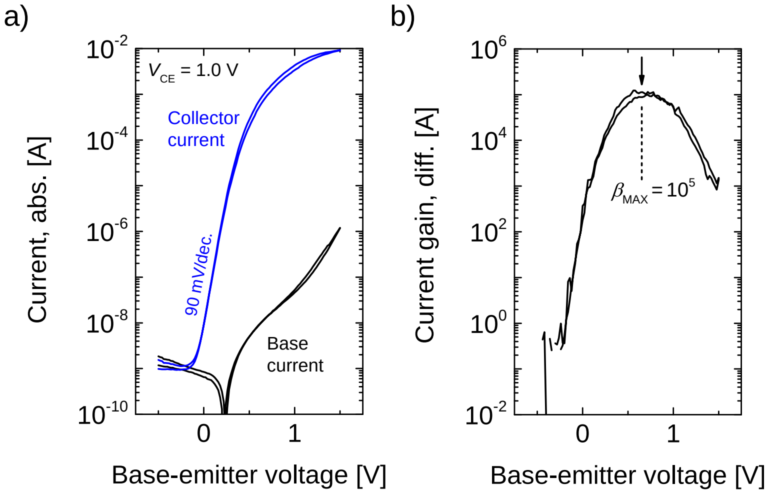

Figure 1: a) Base sweep of an OPBT at an operation voltage of 1.0 V including the base current. b) A maximum differential gain β_{MAX} greater than 10^5 is shown.

2. Calculation of the effective transit length

As motivated in the manuscript, an OPBT acts as two SCLC devices in series. Starting from the Mott-Gurney Law

$$j = \frac{9}{8}\epsilon\epsilon_0\mu\frac{V^2}{L^3}\,, \tag{2}$$

the total voltage V_{tot} drop across two devices is

$$V_{\mathrm{tot}} = V_1 + V_2 = \sqrt{\frac{8jL_1^3}{9\epsilon\epsilon_0\mu}} + \sqrt{\frac{8jL_2^3}{9\epsilon\epsilon_0\mu}} \tag{3}$$

with the thicknesses and voltages L_1, L_2, V_1 and V_2 of device 1 and 2, respectively. Equation 3 can be simplified to

$$V_{\mathrm{tot}} = \sqrt{\frac{8j}{9\epsilon\epsilon_0\mu}}\left(\sqrt{L_1^3} + \sqrt{L_2^3}\right) \tag{4}$$

and comparing to Equation 2, the effective transit length

$$L_{\mathrm{eff}} = \left(\sqrt{L_1^3} + \sqrt{L_2^3}\right)^{2/3} \tag{5}$$

can be found as a reduced effective thickness of the OPBT.

3. Electrode resistance

First, OPBTs with an emitter-top-electrode broadness of 200 µm are investigated. The extracted on-state current from the base sweep in Figure 2 a) represents current points in the diagram of the output characteristic, in Figure 2 b). As seen for current densities above $200\,\mathrm{A\,cm^{-2}}$, an electrode resistance R_{el} of $20\,\Omega$ limits the current. At even higher currents of about $500\,\mathrm{A\,cm^{-2}}$ the top electrode starts ripping off at its edges, cf. Figure 2 c). Ergo, the OPBT can drive such a high current, that even the highly conductive aluminum electrode with a thickness of 100 nm is not able to conduct properly. So, OPBTs with broader emitter electrodes (600 µm) are useful for driving higher current densities to reach $1\,\mathrm{kA\,cm^{-2}}$.

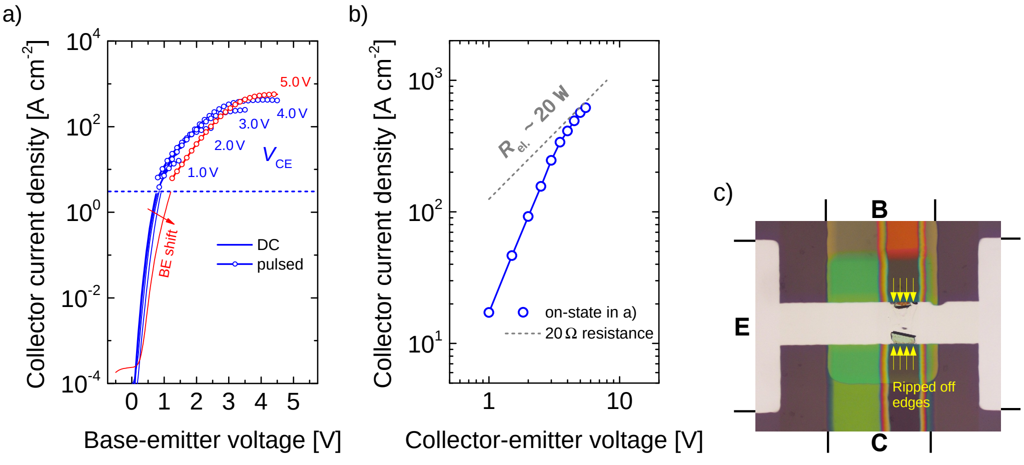

Figure 2: Base sweeps for an OPBT with a narrowed top electrode. Extracting the SCLC-caused saturation points of a) into the output characteristic b), a linear limitation of $20\,\Omega$ occurs representing the resistance R_{el} of the top electrode. c) At high currents above $500\,\mathrm{A\,cm^{-2}}$, the top electrode (emitter) rips off at the edges.

4. Time-resolved pulsed measurement

In Figure 3 a), the pulsed measurement of the OPBT (top 100 nm / bottom 100 nm) is investigated using an oscilloscope. As seen, the collector voltage (blue) encapsulates the base voltage (gray). The pulse measurement takes place in the last 50 % of the combined pulse. The operation voltage (1) and the voltage between the OPBT and a shunt R_{shunt} of 100 Ω are measured for extracting the driven current. According to the measured voltage drop above 0.75 V across the 100 Ω shunt, a collector current of about 19 A cm^{-2} is measured for a V_{BE} of 1.5 V and a V_{CE} of 2.0 V.

In Figure 3 b), the time-resolved current, extracted from the shunt, is shown for different voltages up to a V_{BE} of 3.5 V and a V_{CE} of 6.0 V. With increasing voltages, not only the general current flow increase but also the time-dependent increase of the current gets more distinct. This behavior can be attributed to the self-heating effect, already taking place in a time period of 100µs. So, even at very short pulses of 250 µs, the high power input leads to an electrothermal feedback [1].

In Figure 3 c), the high current density j of 1 kA cm^{-2} is proved once more. Here, a 5 W shunt of 2.5 Ω is used because an input power above 1 W is expected. At a collector-emitter voltage of 12 V and a pulsed base-emitter voltage of almost 7 V, the voltage drop across the shunt reaches values greater then 1 V, which corresponds to a collector current density of 1 kA cm^{-2}.

By that, the transistor can withstand a power dissipation of more than 4 W in 250 µs. The missing time-dependent slope of the current due to self-heating is likely due to bias stress effect which is also be seen to effect base potential varying during the dual pulse measurement.

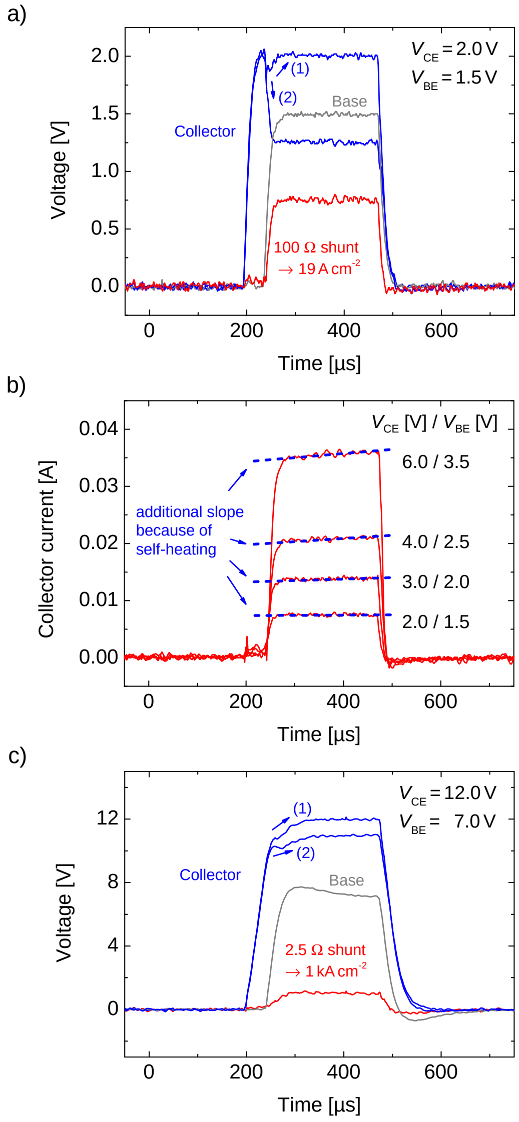

Figure 3: Time-resolved dual pulse measurement. The driving voltage (1) encapsulates the base-emitter voltage (2). Using an oscilloscope, the drop over a shunt connected to the collector, is measured to extract the driven collector current. a) Pulse-measurement with V_{BE} (gray), the two V_{CE} (blue) before (1) and after (2) the shunt of $100\,\Omega$, and the extracted voltage drop (red). b) Extracted currents for different voltages. By increasing the voltage, an increased linear slope during the pulse-time (blue) is observed. c) Using a high-power shunt of $2.5\,\Omega$, the current density of $1\,\mathrm{kA\,cm^{-2}}$ is confirmed.

5. Measurement of transit frequency

As seen in Figure 4, the transit frequency f_T is calculated, extrapolating the decrease of the small-signal curren gain ($|h_{21}|$) with slope of -20 dB dec^{-1} and taking the interception when $|h_{21}|$ is one, while collector and emitter are AC ground. This measurement was done using the characterization setup reported in [2]. For a DC bias current of 100 µA, an f_T of 330 kHz can be measured, whereas at a high current of 6 mA a value of 5 MHz is reached. So, increasing the current results in higher speed due to the higher transconductance of the device. A maximum f_T of about 10 MHz is extrapolated from a single point-measurement reaching a $|h_{21}|$ of 20 at 500 kHz.

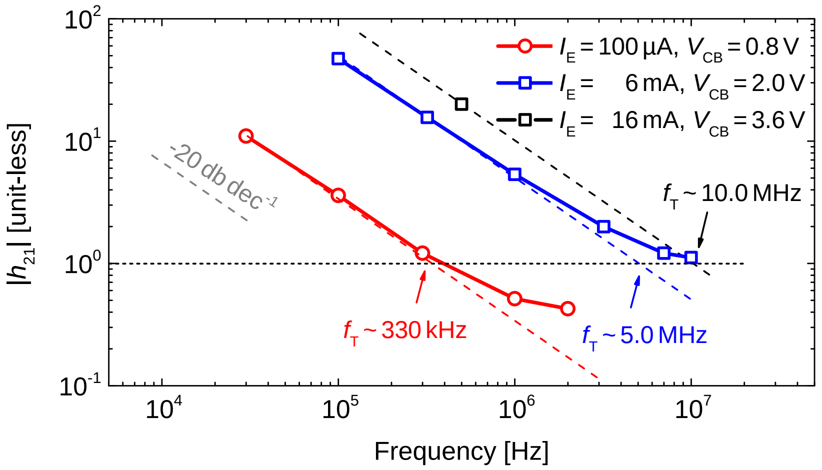

Figure 4: Measurement of the transit frequency f_T: small signal current gain $|h_{21}|$ vs. frequency. f_T is extracted from the interception of the decrease of -20 deb dec.$^{-1}$ and the line when $|h_{21}|$ is equal to one. As seen, injecting a higher current, a higher speed is reached (cf. blue, red & black).

6. Oscillator setup

The circuit and parameters used for the oscillation measurement are shown in Figure 5 and Table 1. The 22 nF capacitor at the base keeps this node on AC ground. In order to have large output swing, the device is DC biased at a collector-base voltage of V_{CB} of $V_{\mathrm{DD}} \times R_{\mathrm{B1}}/(R_{\mathrm{B1}} + R_{\mathrm{B2}})$ larger than 2 V. The bias current is set by the single resistor R_{E} instead of an active current mirror. This makes the circuit quite simple, however, R_{E} could increase the resistive load at v_{e} and therefore deteriorates the quality factor of the L-C tank. This is especially problematic at high bias currents where R_{E} is small. Therefore, a large series L_{E} is added to AC-block R_{E} at the oscillation frequency.

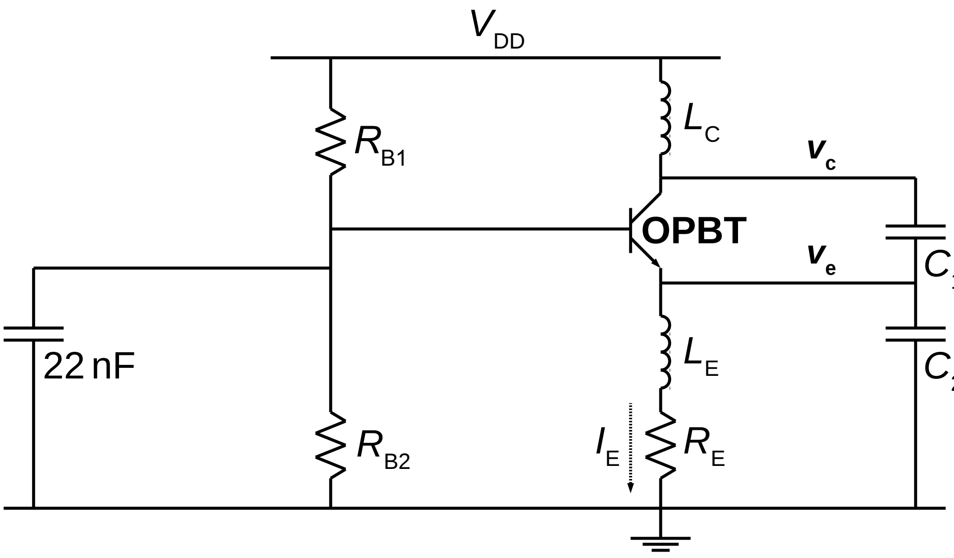

Figure 5: Circuit for testing an OPBT in an oscillator setup. Discrete R-L-C components are used as summarized in Table 1.

[1] A. Fischer, P. Pahner, B. Lüssem, K. Leo, R. Scholz, T. Koprucki, K. Gärtner, A. Glitzky, Self-heating, bistability, and thermal switching in organic semiconductors, Physical Review Letters 110 (12) (2013) 126601. doi:10.1103/PhysRevLett.110.126601.

[2] B. Kheradmand-Boroujeni, G. C. Schmidt, D. Höft, K. Haase, M. Bellmann, K. Ishida, R. Shabanpour, T. Meister, C. Carta, A. C. Hübler, F. Ellinger, Small-signal characteristics of fully-printed high-current flexible all-polymer three-layer-dielectric transistors, Organic Electronics 34 (2016) 267–275. doi:10.1016/j.orgel.2016.04.037.

Parameter	$f_{osc} = 1.87\,\mathrm{MHz}$	$f_{osc} = 3.0\,\mathrm{MHz}$	$f_{osc} = 5.22\,\mathrm{MHz}$
L_C	33 µH	10 µH	5 µH
C_1	100 pF	220 pF	100 pF
C_2	220 pF	220 pF	120 pF
R_{B1}	27 kΩ	18 kΩ	2.7 kΩ
R_{B2}	27 kΩ	27 kΩ	4.7 kΩ
R_E	820 Ω	820 Ω	121 Ω
L_E	0 µH	0 µH	33 µH
V_{DD}	4.22 V	7.1 V	7.37 V
I_E	951 µA	3.2 mA	20.0 mA

Table 1: Summarized *R-L-C* components of Figure 5 for testing an OPBT in an oscillator setup at different frequencies of 1.87 MHz, 3.0 MHz and 5.22 MHz.
